# The Beneficial Effects of P2X_7_ Antagonism in Rats with Bile Duct Ligation-induced Cirrhosis

**DOI:** 10.1371/journal.pone.0124654

**Published:** 2015-05-01

**Authors:** Hung-Chun Tung, Fa-Yauh Lee, Sun-Sang Wang, Ming-Hung Tsai, Jing-Yi Lee, Teh-Ia Huo, Hui-Chun Huang, Chiao-Lin Chuang, Han-Chieh Lin, Shou-Dong Lee

**Affiliations:** 1 Institute of Pharmacology, National Yang-Ming University School of Medicine, Taipei, Taiwan; 2 Faculty of Medicine, National Yang-Ming University School of Medicine, Taipei, Taiwan; 3 Division of Gastroenterology, Department of Medicine, Taipei Veterans General Hospital, Taipei, Taiwan; 4 Division of General Medicine, Department of Medicine, Taipei Veterans General Hospital, Taipei, Taiwan; 5 Department of Medical Affair and Planning, Taipei Veterans General Hospital, Taipei, Taiwan; 6 Division of digestive therapeutic endoscopy, Chang Gung Memorial Hospital, Taipei, Taiwan; 7 Chang Gung University College of Medicine, Taoyuan, Taiwan; 8 Division of Gastroenterology, Department of Medicine, Cheng Hsin General Hospital, Taipei, Taiwan; University of Navarra School of Medicine and Center for Applied Medical Research (CIMA), SPAIN

## Abstract

Splanchnic angiogenesis in liver cirrhosis often leads to complications as gastroesophageal variceal hemorrhage and the treatment efficacy is adversely affected by poor portal-systemic collateral vasoresponsiveness related to nitric oxide (NO). Purinergic receptor subtype P2X_7_ participates in the modulation of inflammation, angiogenesis, fibrogenesis and vasoresponsiveness, but the relevant influence in cirrhosis is unknown. Common bile duct-ligated (CBDL) or sham-operated Spraque-Dawley rats received brilliant blue G (BBG, a P2X_7_ antagonist and food additive) or vehicle from the 15^th^ to 28^th^ day after operations, then hemodynamics, mesenteric angiogenesis, portal-systemic shunting, liver fibrosis, and protein expressions of angiogenic and fibrogenic factors were evaluated. The influence of oxidized ATP (oATP, another P2X_7_ receptor antagonist) on the collateral vasoresponsiveness to arginine vasopressin (AVP) was also surveyed. BBG decreased superior mesenteric artery (SMA) flow, portal-systemic shunting, mesenteric vascular density, and mesenteric protein expressions of vascular endothelial growth factor (VEGF), VEGF receptor 2 (VEGFR2), phospho (p)-VEGFR2, platelet-derived growth factor (PDGF), PDGF receptor beta (PDGFRβ), cyclooxygenase (COX)-1, COX-2, and endothelial NO synthase (eNOS) in CBDL rats. BBG also ameliorated liver fibrosis and down-regulated hepatic interleukin-6 (IL-6), tumor necrosis factor-alpha (TNF-α), PDGF, IL-1β, transforming growth factor-beta (TGF-β), p-extracellular-signal-regulated kinases (ERK), and alpha-smooth muscle actin (α-SMA) expressions in CBDL rats. The collateral vasocontractility to AVP was enhanced by oATP. oATP down-regulated eNOS, inducible NOS (iNOS), VEGF, Akt, p-Akt, and nuclear factor-kappa B (NF-κB) expressions in splenorenal shunt, the most prominent intra-abdominal collateral vessel in rodents. P2X_7_ antagonism alleviates splanchnic hyperemia, severity of portal-systemic shunting, mesenteric angiogenesis, liver fibrosis, and enhances portal-systemic collateral vasoresponsiveness in cirrhotic rats. P2X_7_ blockade may be a feasible strategy to control cirrhosis and complications.

## Introduction

Liver inflammation is usually followed by fibrogenesis, cirrhosis and ultimately, complications from increased intrahepatic resistance and portal hypertension. Portal-systemic collateral vessels develop during the course and bleeding from the collaterals, especially the gastroesophageal varices, and is one of the most dreadful complications among cirrhotic patients. Angiogenesis plays a role in the process [1] and even worse, the poor vasoresponsiveness to vasoconstrictors during acute hemorrhage adversely influences the treatment efficacy [2].

Liver cirrhosis with portal hypertension is characterized by systemic and splanchnic vasodilatory substances release, especially nitric oxide (NO) and prostacyclin [3], which lead to hyperdynamic circulatory status, and further increased mesenteric blood flow and portal inflow. NO and prostacyclin are synthesized by NO synthases (NOS, inducible (iNOS) and endothelial (eNOS) forms) and cyclooxygenases (COX, COX-1 and COX-2), respectively. We have demonstrated that NO and/or prostacyclin inhibition enhanced the collateral vasoresponsiveness to arginine vasopressin (AVP) in portal hypertensive rats [4], suggesting their role in this situation. A previous study, on the other hand, indicated that NO participates in the portal hypertensive angiogenesis [5]. Increased vascular endothelial growth factor (VEGF), VEGF receptor 2 (VEGFR2), and CD31 (endothelial cell marker) expressions in mesentery of portal hypertensive mice have also been found, which provided the evidence of mesenteric angiogenesis in portal hypertension [1]. Inhibition of VEGFR2 attenuated hyperdynamic splanchnic circulation and collaterals in portal hypertensive rats, suggesting the beneficial effects of anti-angiogenesis in ameliorating this pathological condition [6].

Lipopolysaccharide (LPS) and bacterial breakdown products in the gastrointestinal tract reach the liver via portal vein. The hepatic microenvironment is thus unique by the presence of bacterial endotoxin and the elicited mediators. ATP released from damaged cells as a result of inflammation serves as a cell-to-cell mediator through cell surface P2 purinergic receptors [7]. Among the purinergic receptors, P2X_7_ receptor has been noticed for its role in the release of pro-inflammatory mediators: ATP induces cytokine release through P2X_7_ receptor in hemopoietic cells. In mice lacking P2X_7_ receptors, peritoneal macrophages failed to release interleukin-1β (IL-1β) in response to ATP [8]. It is noteworthy that a P2X_7_ agonist 2',3'-(4-benzoyl) benzoyl ATP (BzATP) induced tumor necrosis factor (TNF) release much more effectively than ATP [9]. In addition, P2X_7_ receptors activation in the presence of LPS stimulated iNOS expression and NO production, which elicited vasodilation [10].

Brilliant blue G (BBG), a commonly used blue dye as food additive without significant toxicity, noncompetitively inhibits the P2X_7_ receptor and is currently the most potent P2X_7_ receptor antagonist in rats [11]. Administration of BBG 15 min after thoracic spinal cord injury in rats alleviated spinal cord damage via ameliorating local inflammation, which was evidenced by reduced neutrophil infiltration [12]. BBG also significantly inhibited ATP-BzATP-induced TNF release [13]. Another agent without the dying effect, oxidized ATP (oATP), completely and irreversibly antagonized P2X_7_ receptor on macrophage [14]. oATP also attenuated LPS-induced COX-2 [15] and iNOS [16] over-expressions in murine macrophages, and IL-1β secretion from murine microglial cells [17]. Furthermore, oATP inhibited angiogenesis by suppression of matrix metalloproteinases (MMP)-2, MMP-9, and VEGF production in a hindlimb-ischemic mouse model [18].

Collectively, P2X_7_ antagonism ameliorates inflammation, down-regulates NOS, COX and proangiogenic factors expressions and inhibits angiogenesis as well as vasodilation, but the relevant investigation on liver cirrhosis has not been performed. In this study, we aimed to survey the effects and mechanisms of selective P2X_7_ antagonism on liver fibrosis, hemodynamics, mesenteric angiogenesis, severity and vasoconstrictor responsiveness of portal-systemic collaterals in rats with common bile duct ligation (CBDL)-induced cirrhosis.

## Materials and Methods

### Animal model

Male Sprague-Dawley rats (260–280 g) were caged at 24°C with a 12 h light/dark circle and allowed free access to food and water. Secondary biliary cirrhosis was induced by CBDL [19]. In brief, under ketamine anesthesia (100 mg/kg, intramuscularly), the common bile duct was exposed through a midline abdominal incision, and doubly ligated with 3–0 silk. The section between the ligatures was cut. The incision was then closed and the animal was allowed to recover. A high yield of secondary biliary cirrhosis was noted four weeks later [19, 20]. Weekly vitamin K injection (50 μg/kg, intramuscularly) was applied to avoid coagulation defect. This study had been approved by Taipei Veterans General Hospital Animal Committee (IACUC98-170). The Guides for the Care and Use of Laboratory Animals (NIH publication no. 85–23, rev. 985, U.S.A.) were followed.

### Systemic and portal hemodynamics

The right carotid artery was cannulated with PE-50 catheters, and the mesenteric vein with 18-gauge Teflon cannula. They were connected to a Spectramed DTX transducer (Spectramed Inc., Oxnard, CA, U.S.A.). The external zero reference was set at the level of the mid-portion of the animal. Recordings of mean arterial pressure (MAP), heart rate (HR), and portal pressure (PP) were performed [21]. Superior mesenteric artery (SMA) flow was measured by a non-constrictive perivascular ultrasonic transit-time flow probe (lRB, 1-mm diameter; Transonic Systems, Ithaca, NY, U.S.A.), expressed as mL/min per 100 g body weight [22].

### Portal-systemic shunting determination

Portal-systemic shunting was evaluated as previously described [23] with a slight modification. The color microspheres substituted for radioactive microspheres. Briefly, 30,000 of 15-μm yellow microspheres (Dye Track; Triton Technology, San Diego, CA, U.S.A.) were slowly injected into the spleen. The rats were euthanized, and then the liver and lung were dissected. The number of microspheres was determined following the protocol provided by the manufacturer: 3,000 blue microspheres served as internal control. Spillover between wavelengths was corrected with the matrix inversion technique. Portal-systemic shunting was calculated as (microspheres): lung/(liver plus lung). Assuming a worst-case scenario in which two-thirds of the microspheres remain trapped in the spleen, this technique can detect a minimum shunt of 3.5%.

### *In situ* portal-systemic collateral perfusion

As previously described [4], both jugular veins were cannulated with 16-gauge Teflon cannulas as outlets of perfusate. The inlet is an 18-gauge Teflon cannula inserted in the superior mesenteric vein. To exclude the liver from perfusion, the portal vein was tied. The animal was transferred into a chamber (37±0.5°C). Perfusion was started via the mesenteric cannula by a roller pump (model 505S; Watson-Marlow Limited, Falmouth, Cornwall, UK) with Krebs solution equilibrated with 95% (v/v) O_2_ and 5% (v/v) CO_2_ by a silastic membrane lung [24]. Pneumothorax was created by opening slits through the diaphragm to increase the pulmonary arterial resistance and to prevent the perfusate from entering the left heart. Experiments were performed 25 min after starting perfusion at a constant rate of 12 ml/min. As the flow rate was kept constant, the measured perfusion pressure reflected collateral vascular resistance. Only one concentration-response curve was performed in each preparation and the contracting capability was challenged with a 125 mM potassium chloride solution at the end.

### Liver biochemistry

Blood was centrifuged at 3,000 g for 10 min then the supernatant was collected to measure the concentrations of aspartate transaminase (AST), alanine transaminase (ALT), and total bilirubin using the analyzer (Cobas C501, Roche, U.S.A.).

### Histology

Five micrometer-thick sections obtained from paraffin-embedded livers were stained with Hematoxylin and Eosin (H&E) for the evaluation of structural changes, with Sirius red for fibrosis (collagen deposition), and with alpha-smooth muscle actin (α-SMA) for hepatic stellate cell (HSC) activation. The immunohistochemical staining was modified from the previous study [25]. In brief, liver sections were de-waxed with xylene and rehydrated through a series of ethanol solutions with different concentrations. Sections were subjected to microwave irradiation in citrate buffer to enhance antigen retrieval and pre-incubated with 5% normal rabbit serum in Tris-buffered saline. Primary antibody (anti-α-SMA rabbit polyclonal antibody [Abcam, Cambridge, MA, U.S.A.]) was incubated for 1 h in a humidity chamber. After rinsing twice in phosphate buffered saline, sections were incubated with fluorochrome conjugated secondary antibody (Alexa Fluor 488 fluorescent, Jackson ImmunoResearch Laboratories, Inc. Baltimore, USA) for 1 h at room temperature. Finally, slides were coverslipped with carbonate-buffered glycerol, and evaluated in an Olympus AX 80 microscope (Olympus; Hamburg, Germany) equipped with epifluorescence illumination and digital cameras. Image J software (downloaded from the National Institutes of Health (http://rsb.info.nih.gov/ij/)) was used to measure the percentage of Sirius red-stained area. Briefly, grayscale image was used, then the red area was identified using thresholding function. The thresholded area was measured and shown as the percentage of thresholded area per image [26].

### Immunofluorescent study for mesenteric angiogenesis

Mesenteric angiogenesis was quantified by CD31-labelled microvascular networks in rat mesenteric windows according to the previous study with a slight modification [26]. From each rat, at least four mesenteric windows were dissected free, washed in PBS, dried on gelatin slides, and fixed in 100% MeOH (-20°C for 30 min). Slides were then incubated overnight at 4°C with the primary antibody mouse anti-rat CD31-biotin (AbD Serotec, Oxford, UK). Then secondary antibody (CY2-conjugated streptavidin; Jackson ImmunoResearch, West Grove, PA, U.S.A.) was applied for 1 h at room temperature. (×100)-magnification immunofluorescent images were assessed using an upright fluorescent microscope (AX80, Olympus, Japan) and thresholded by Image J. The vascular length was manually measured with the pencil tool and the vascular area automatically with histogram function, respectively. According to the information provided by QICAM, with the eyepiece 10X, the diameter of one pixel on an image taken with QICAM equals to 4.65 μm. Under 100X-magnification (objective 10X and eyepiece 10X), the diameter of one pixel would be 4.65 μm/10 = 0.465 μm. The vascular length could thus be determined accordingly. The unit of vascular length per unit area of mesenteric window would be μm•(μm^2^)^-1^ = μm^-1^ and the vascular area per unit area of mesenteric window, could be pixel•pixel^-1^ without being converted to μm^2^•(μm^2^)^-1^.

### Western blot analysis of mesentery, liver, and splenorenal shunt

Microfuge tubes containing protein extraction solution and grounded samples were spun at 10,000 g for 10 min (4°C). The supernatant was stored at -80°C. Protein concentration was measured by a protein assay kit (Bio-Rad, Hercules, CA, U.S.A.). Protein (20 μg) was resolved by SDS-PAGE and transferred to a PVDF membrane (Bio-Rad). The membrane was blocked with 5% non-fat milk for 1 h at room temperature, and then incubated overnight at 4°C in primary antibody: anti-VEGF rabbit polyclonal antibody (Gene Tex, Irvine, U.S.A.); anti-VEGFR2 rabbit polyclonal antibody (Cell signaling, Danvers, MA, U.S.A.); anti-p-VEGFR2 rabbit polyclonal antibody (Upstate, New York, U.S.A.); anti-platelet-derived growth factor (PDGF) rabbit polyclonal antibody (Santa Cruz, California, U.S.A.); anti-PDGF receptor beta (PDGFRβ) mouse monoclonal antibody (Cell signaling); anti-COX-1 rabbit polyclonal antibody (Upstate); anti-COX-2 rabbit polyclonal antibody (Abcam); anti-eNOS rabbit monoclonal antibody (Cell signaling); anti-iNOS rabbit polyclonal antibody (Millipore, CA, U.S.A.); anti-Akt rabbit monoclonal antibody (Cell signaling); anti-p-Akt rabbit polyclonal antibody (Cell signaling); anti- nuclear factor-kappa B (NF-κB) rabbit polyclonal antibody (Santa Cruz); anti-transforming growth factor-beta (TGF-β) mouse monoclonal antibody (Abcam); anti-α-SMA rabbit polyclonal antibody (Abcam); anti-Smad2 rabbit monoclonal antibody (Abcam); anti-p-Smad2 rabbit polyclonal antibody (Millipore); anti-Smad3 rabbit monoclonal antibody (Millipore); anti-p-Smad3 rabbit polyclonal antibody (Millipore); anti-Smad7 rabbit polyclonal antibody (Abcam); anti-extracellular signal-regulated kinase (ERK) mouse monoclonal antibody (Millipore); anti-p-ERK rabbit monoclonal antibody (Cell signaling); anti-IL-6 rabbit monoclonal antibody (Thermo Fisher Scientific, MA, U.S.A.); anti-TNF-α rabbit monoclonal antibody (Cell signaling); anti-IL-1β rabbit oligoclonal antibody (Thermo Fisher Scientific). β-actin expression served as loading control. After that, the membrane was washed and incubated with secondary antibody: anti-rabbit antibody (Chemicon, Shinagawa-ku, Tokyo, Japan) or anti-mouse antibody (Chemicon) for 1 h at room temperature then washed. The blots were analyzed using a computer-assisted video densitometer and digitalized software (Kodak Digital Science ID Image Analysis Software, Eastman Kodak Co., U.S.A.).

### Study protocol

#### Chronic treatment on CBDL or sham rats

CBDL or sham rats received either BBG (100 mg/kg, p.o., CBDL-BBG, sham-BBG) or vehicle (saline, CBDL-vehicle, sham-vehicle) since the 15^th^ day to 28^th^ day after operations. On the 29^th^ day, two series of experiments were performed: First, portal-systemic shunting determination; Second, after body weight (BW) and hemodynamic (MAP, PP, HR) measurements, liver, mesentery, mesenteric window were collected to survey the severity of liver fibrosis, mesenteric angiogenesis and expressions of fibrotic and angiogenic factors. Blood was collected for biochemistry survey.

#### Acute treatment on CBDL rats

On the 29^th^ day after CBDL, with an *in situ* collateral perfusion model, rats were randomly allocated to receive pre-incubation with Krebs solution (vehicle control) or oATP (specific P2X_7_ antagonist, 50 μM) since 1 h prior to and throughout the perfusion experiments. The pre-incubation time of oATP was decided according to the previous study [10], showing that pretreatment of aortas with oATP for 1 hour inhibited the augmented LPS plus BzATP-induced iNOS protein expression. One h after oATP pre-incubation, AVP was added to the perfusate at a 3-minute interval for each escalating concentration (10^-10^, 10^-9^, 3 × 10^-9^, 10^-8^, 3 × 10^-8^, 10^-7^ M) and cumulative concentration-response curves of portal-systemic collateral vascular bed to AVP were obtained. Splenorenal shunts were collected at the end of perfusion experiments.

### Drugs

AVP, BBG, oATP and reagents for Krebs solution were purchased from Sigma Chemical Co. (St. Louis, Mo., U.S.A.). All solutions were freshly prepared on the days of experiments.

### Statistical Analyses

All results are reported as mean ± standard error of mean (S.E.M.). An unpaired Student *t* test or one-way analysis of variance (ANOVA) with LSD’s post-hoc test was used for the determination of statistical significance between or among experimental groups. A P value < 0.05 denotes the level of significance.

## Results

### Chronic BBG treatment

#### Body weight and hemodynamics

Table 1 depicts the BW and hemodynamics in sham or CBDL rats after vehicle or BBG treatment. BBG did not affect sham rats but significantly decreased SMA flow in CBDL rats (CBDL-vehicle vs. CBDL-BBG: p = 0.008).

**Table 1:** Body weight and hemodynamic effects in sham or CBDL rats treated with vehicle or BBG.

	sham-vehicle *n* = 9	sham-BBG *n* = 10	CBDL-vehicle *n* = 8	CBDL-BBG *n* = 8
**BW (g)**	432.0±5.3	430.6±6.6	367.5±6.7^a^ ^b^	382.0±9.2^a^ ^b^
**MAP (mm Hg)**	126.9±3.1	125.4±3.5	93.7±5.1^a^ ^b^	84.2±4.5^a^ ^b^
**HR (beats/min)**	354.8±16.2	366.6±24.3	278.9±11.7^a^ ^b^	244.8±7.6^a^ ^b^
**PP (mm Hg)**	7.8±0.3	8.4±0.4	16.7±1.1^a^ ^b^	15.4±1.2^a^ ^b^
**SMA flow (mL/min/100 g)**	3.2±0.2	3.7±0.3	6.2±0.5^a^ ^b^	4.8±0.3^a^ ^b^ ^**c**^

BW: body weight; MAP: mean arterial pressure; HR: heart rate; PP: portal pressure; SMA: superior mesenteric artery

^**a**^ *P*<0.01 vs. sham-vehicle group.

^**b**^ *P*<0.05 vs. sham-BBG group.

^**c**^ *P*<0.01 vs. CBDL-vehicle group.

#### Liver biochemistry

Table 2 demonstrates that BBG did not affect sham rats but significantly lowered ALT level in CBDL rats (CBDL-vehicle vs. CBDL-BBG, p = 0.002).

**Table 2:** Liver biochemistry in sham or CBDL rats treated with vehicle or BBG.

	sham-vehicle *n* = 8	sham-BBG *n* = 8	CBDL-vehicle *n* = 7	CBDL-BBG *n* = 8
**AST (U/L)**	139.0±14.0	118.1±11.3	795.4±71.7^a^ ^b^	746.3±95.6^a^ ^b^
**ALT (U/L)**	57.9±5.0	50.0±6.3	188.6±18.7^a^ ^b^	132.1±11.8^a^ ^b^ ^**c**^
**Total bilirubin (mg/dL)**	0.7±0.0	0.7±0.0	6.8±0.3^a^ ^b^	6.9±0.3^a^ ^b^

AST: aspartate transaminase; ALT: aspartate transaminase

^**a**^ *P*<0.01 vs. sham-vehicle group.

^**b**^ *P*<0.01 vs. sham-BBG group.

^**c**^ *P*<0.05 vs. CBDL-vehicle group.

#### Portal-systemic shunting ratio and mesenteric vascular density

**Fig 1:**
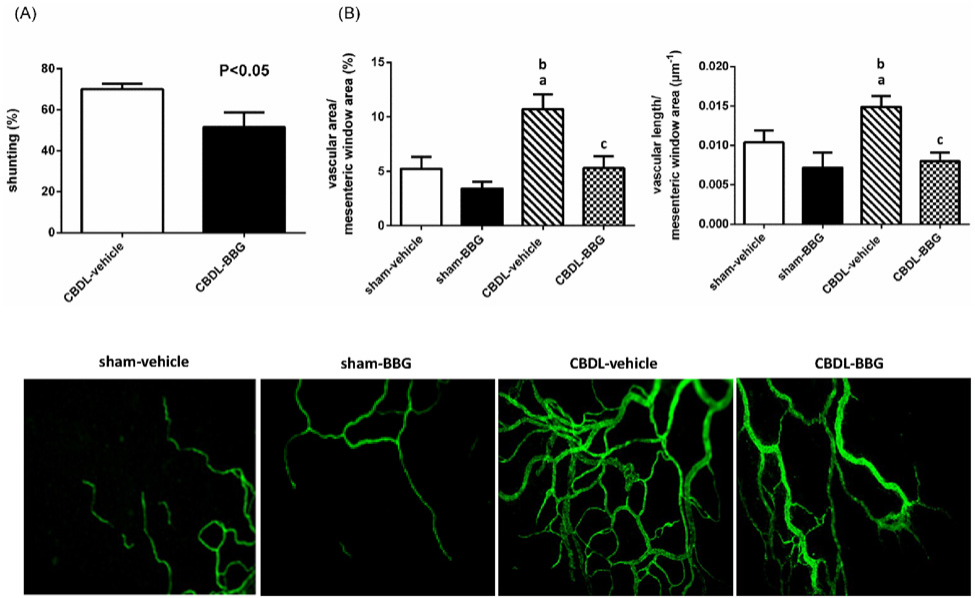
**(A) Portal-systemic shunting in common bile duct-ligated (CBDL) rats treated with vehicle or brilliant blue G** (BBG, CBDL-vehicle: n = 5; CBDL-BBG: n = 7). Compared with vehicle, BBG significantly improved the severity of shunting. **(B) Mesenteric vascular density in sham or CBDL rats treated with vehicle or BBG** (sham-vehicle: n = 6; sham-BBG: n = 6; CBDL-vehicle: n = 6; CBDL-BBG: n = 9). Vascular area and length per unit of mesenteric window area were significantly reduced in CBDL rats with BBG treatment. ^a^p<0.05 vs. sham-vehicle group. ^b^p<0.005 vs. sham-BBG group. ^c^p<0.005 vs. CBDL-vehicle group.

The severity of portal-systemic shunting in rats with vehicle or BBG treatment is shown in Fig 1(A). Compared with vehicle-treated CBDL rats, BBG significantly alleviated the severity of shunting (CBDL-vehicle vs. CBDL-BBG [%]: 70.1±2.6 vs. 51.6±7.0, p = 0.041). Fig 1(B) depicts that CBDL markedly elevated mesenteric vascular density as evidenced by CD31 immunofluorescence (sham-vehicle vs. CBDL-vehicle: vascular area per unit window area [%]: 5.2447±1.0542 vs. 10.7058±1.3623, p = 0.003; vascular length per unit area [μm^-1^x100]: 0.0104±0.0015 vs. 0.0149±0.0014, p = 0.047). Furthermore, BBG significantly decreased the mesenteric vascular density in CBDL rats (CBDL-vehicle vs. CBDL-BBG: vascular area ratio: 10.7058±1.3623 vs. 5.2932±1.0874, p = 0.001; vascular length ratio: 0.0149±0.0014 vs. 0.0080±0.0011, p = 0.002). There was no significant difference between sham rats with or without BBG treatment (sham-vehicle vs. sham-BBG: vascular area ratio [%]: 5.2447±1.0542 vs. 3.4313±0.5991, p = 0.279; vascular length ratio [μm^-1^x100]: 0.0104±0.0015 vs. 0.0072±0.0019, p = 0.158).

#### Mesenteric angiogenic factor protein expressions

**Fig 2:**
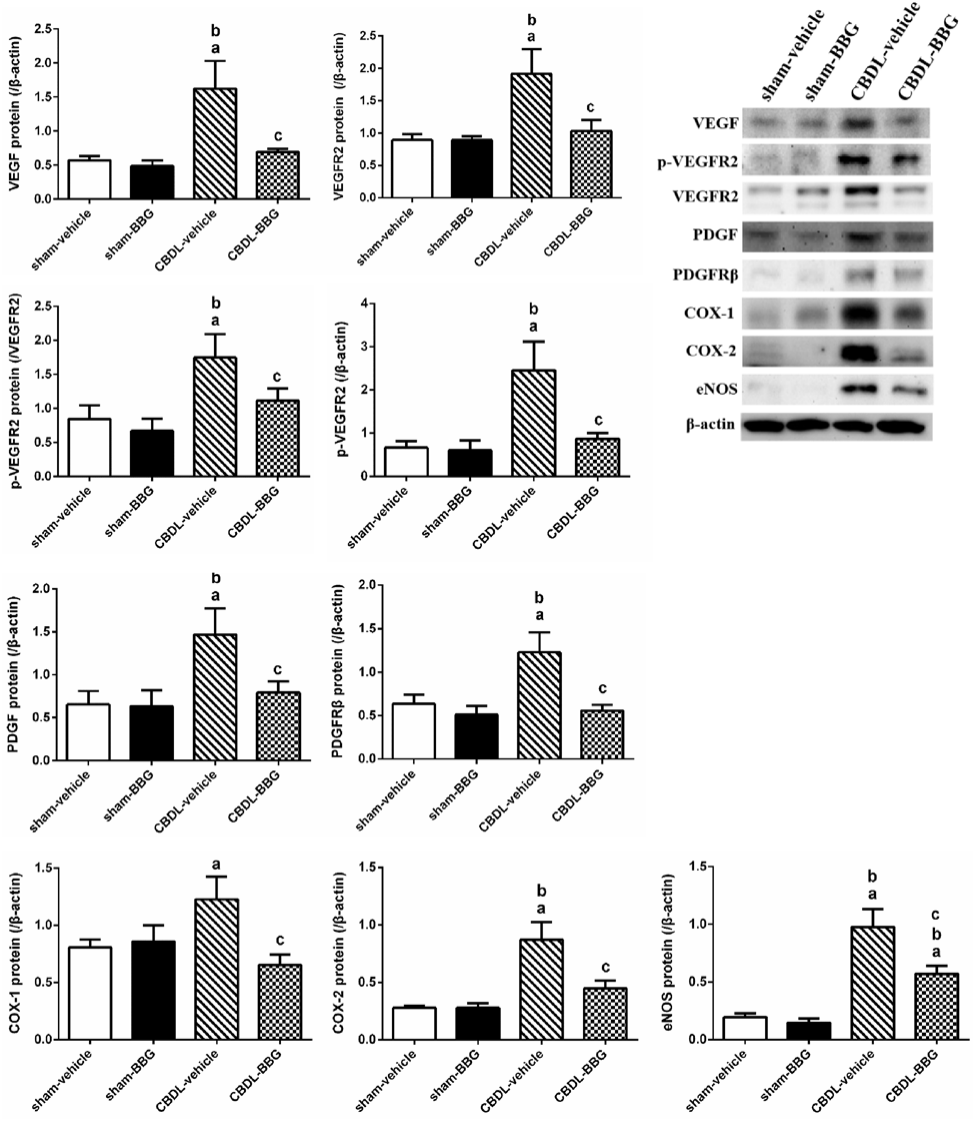
Mesenteric VEGF, VEGFR2, p-VEGFR2, PDGF, PDGFRβ, COX-1, COX-2, and eNOS protein expressions in sham or common bile duct-ligated (CBDL) rats treated with vehicle or brilliant blue G (BBG, sham-vehicle: n = 7; sham-BBG: n = 7; CBDL-vehicle: n = 6; CBDL-BBG: n = 6). BBG significantly down-regulated the mesenteric angiogenic factor expressions in CBDL rats. ^**a**^p<0.05, vs. sham-vehicle group. ^**b**^p<0.005 vs. sham-BBG group. ^**c**^p<0.05 vs. CBDL-vehicle group.

Fig 2 demonstrates that BBG did not significantly influence mesenteric VEGF, VEGFR2, p-VEGFR2, PDGF, PDGFRβ, COX-1, COX-2, and eNOS protein expressions in sham rats (sham-vehicle vs. sham-BBG [/β-actin]: VEGF: 0.5691±0.0627 vs. 0.4862±0.0843; VEGFR2: 0.8957±0.0875 vs. 0.8926±0.0602; p-VEGFR2: 0.6571±0.1541 vs. 0.5990±0.2363; PDGF: 0.6523±0.1579 vs. 0.6301±0.1873; PDGFRβ: 0.6351±0.1076 vs. 0.5092±0.1013; COX-1: 0.8066±0.0677 vs. 0.8569±0.1413; COX-2: 0.2783±0.0228 vs. 0.2787±0.0436; eNOS: 0.1939±0.0324 vs. 0.1463±0.0365; [p-VEGFR2/VEGFR2]: 0.8416±0.2022 vs. 0.6675±0.1814, all p>0.05). CBDL resulted in angiogenic factor up-regulations (sham-vehicle vs. CBDL-vehicle [/β-actin]: VEGF: 0.5691±0.0627 vs. 1.6249±0.4056, p = 0.001; VEGFR2: 0.8957±0.0875 vs. 1.9163±0.3809, p = 0.001; p-VEGFR2: 0.6571±0.1541 vs. 2.4508±0.6701, p = 0.001; PDGF: 0.6523±0.1579 vs. 1.4668±0.3049, p = 0.010; PDGFRβ: 0.6351±0.1076 vs. 1.2264±0.2308, p = 0.006; COX-1: 0.8066±0.0677 vs. 1.2282±0.1945, p = 0.037; COX-2: 0.2783±0.0228 vs. 0.8704±0.1555, p<0.001; eNOS: 0.1939±0.0324 vs. 0.9772±0.1537, p<0.001). [p-VEGFR2/VEGFR2]: 0.8416±0.2022 vs. 2.0981±0.3495, p = 0.001). In addition, compared with vehicle, BBG significantly reduced mesenteric angiogenic factors protein expressions in CBDL rats (CBDL-vehicle vs. CBDL-BBG [/β-actin]: VEGF: 1.6249±0.4056 vs. 0.6941±0.0455, p = 0.002; VEGFR2: 1.9163±0.3809 vs. 1.0335±0.1690, p = 0.004; p-VEGFR2: 2.4508±0.6701 vs. 0.8692±0.1345, p = 0.002; PDGF: 1.4668±0.3049 vs. 0.7918±0.1311, p = 0.033; PDGFRβ: 1.2264±0.2308 vs. 0.5560±0.0670, p = 0.003; COX-1: 1.2282±0.1945 vs. 0.6538±0.0916, p = 0.006; COX-2: 0.8704±0.1555 vs. 0.4465±0.0686, p = 0.001; eNOS: 0.9772±0.1537 vs. 0.5708±0.0711, p = 0.003. [p-VEGFR2/VEGFR2]: 2.0981±0.3495 vs. 1.2224±0.1811, p = 0.018).

#### Liver histology

**Fig 3:**
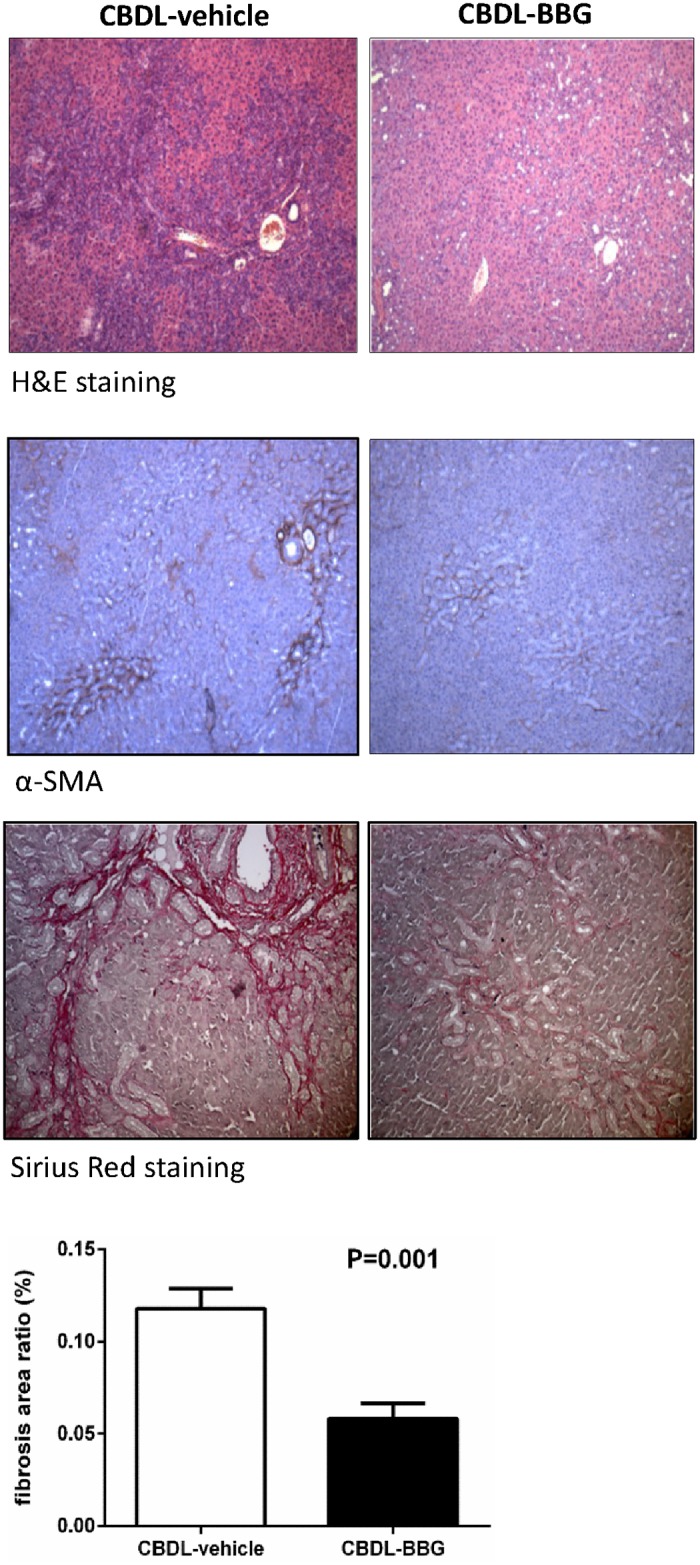
Representative images of hepatic H&E staining, immunohistochemistry staining of α-SMA, and Sirius red staining in common bile duct-ligated (CBDL) rats with vehicle or brilliant blue G (BBG) treatment. CBDL rats treated with BBG had less inflammatory cells infiltration, bridging necrosis, and α-SMA expression than those with vehicle treatment. The degree of collagen fiber deposition was less in CBDL rats with BBG treatment (CBDL-vehicle: n = 6; CBDL-BBG: n = 6).

The H&E staining of the liver from vehicle-treated CBDL rats showed inflammatory cells infiltration with bridging necrosis and fibrotic band interposition, which was ameliorated by BBG. Nevertheless, the bile ductules proliferation was not significantly influenced by BBG. The immunohistochemical staining demonstrated that BBG reduced α-SMA expression. Sirius red staining depicted that the degree of collagen fiber deposition was significantly less in CBDL rats with BBG treatment as compared with those treated with vehicle (CBDL-vehicle vs. CBDL-BBG: fibrosis area ratio [%]: 0.1179±0.0108 vs. 0.0581±0.0084, p = 0.001). The representative images of H&E, immunohistochemistry, and Sirius red staining are shown in Fig 3.

#### Hepatic pro-inflammatory and fibrotic mediator protein expressions

**Fig 4:**
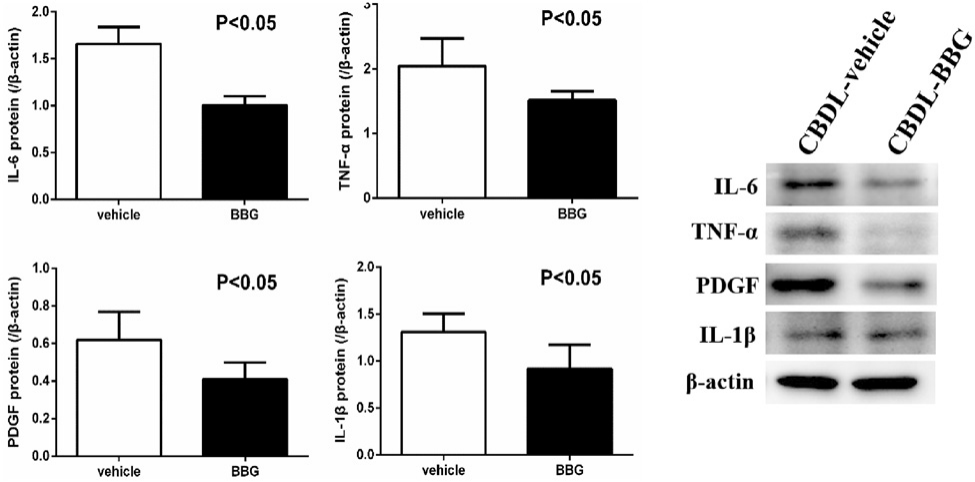
The hepatic IL-6, TNF-α, PDGF, and IL-1β protein expressions in common bile duct-ligated (CBDL) rats with vehicle or brilliant blue G (BBG) treatment (CBDL-vehicle: n = 7; CBDL-BBG: n = 7). BBG down-regulated hepatic IL-6, TNF-α, PDGF, and IL-1β expressions in CBDL rats.

**Fig 5:**
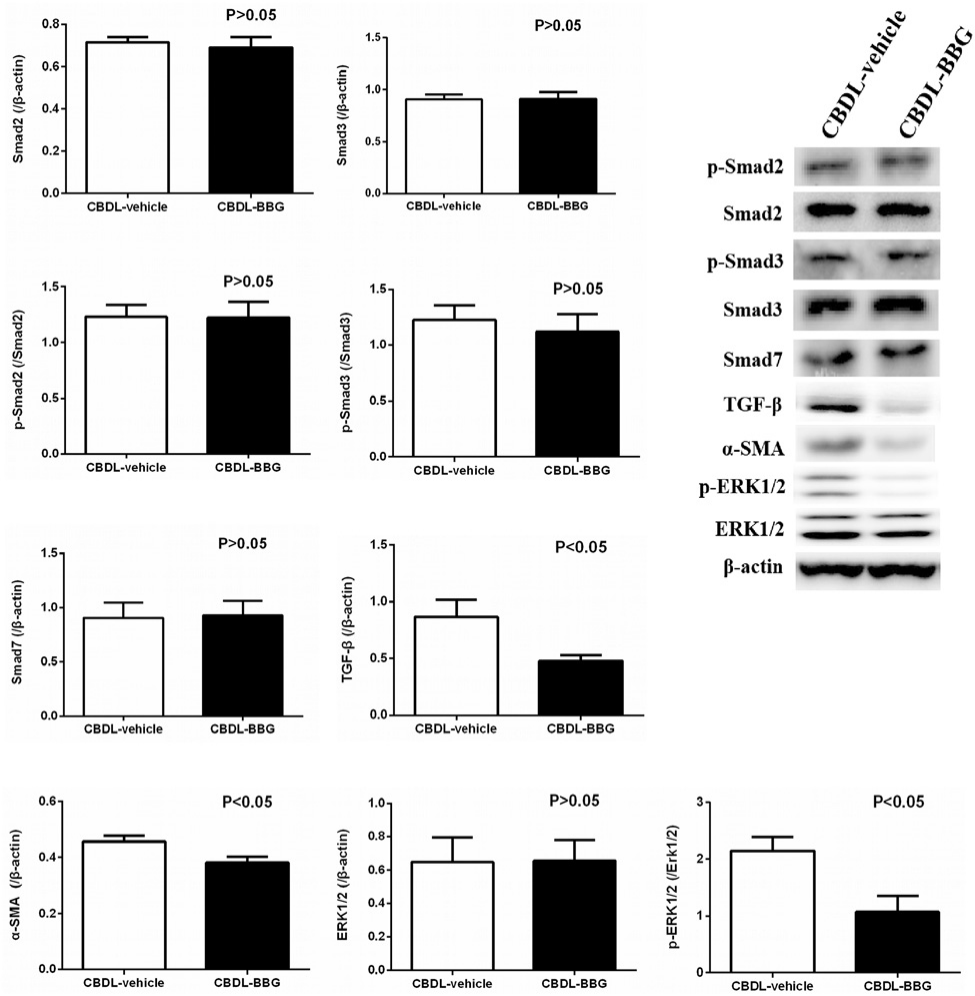
The hepatic Smad2, p-Smad2, Smad3, p-Smad3, Smad7, TGF-β, α-SMA, ERK, and p-ERK/ERK protein expressions in common bile duct-ligated (CBDL) rats with vehicle or brilliant blue G (BBG) treatment (CBDL-vehicle: n = 7; CBDL-BBG: n = 7). BBG did not significantly influence the Smad-signaling factors but down-regulated TGF-β, p-ERK, and α-SMA expressions in livers of CBDL rats.

Fig 4 indicates that BBG down-regulated hepatic IL-6, TNF-α, PDGF, and IL-1β protein expressions in CBDL rats (CBDL-vehicle vs. CBDL-BBG: [/β-actin]: IL-6: 1.6599±0.1794 vs. 1.0037±0.0933, p = 0.012; TNF-α: 2.0433±0.1740 vs. 1.5155±0.0624, p = 0.027; PDGF: 0.6197±0.0667 vs. 0.4096±0.0401, p = 0.027; IL-1β: 1.3079±0.0871 vs. 0.9176±0.1146, p = 0.027). In addition, Fig 5 shows that BBG did not affect hepatic Smad-signaling pathway factors in CBDL rats (CBDL-vehicle vs. CBDL-BBG: [/β-actin]: Smad2: 0.7161±0.0250 vs. 0.6908±0.0507; Smad3: 0.9087±0.0469 vs. 0.9147±0.0648; Smad7: 0.9044±0.1456 vs. 0.9252±0.1374; [p-Smad2/Smad2]: 1.2314±0.1066 vs. 1.2231±0.1410; [p-Smad3/Smad3]: 1.2263±0.1275 vs. 1.1188±0.1597, all p>0.05). Alternatively, BBG significantly down-regulated hepatic TGF-β, p-ERK, and α-SMA protein expressions (CBDL-vehicle vs. CBDL-BBG: [/β-actin]: TGF-β: 0.8646±0.1516 vs. 0.4776±0.0530, p = 0.037; ERK: 0.6471±0.1477 vs. 0.6550±0.1225, p = 0.9673; α-SMA: 0.4568±0.0211 vs. 0.3805±0.0217, p = 0.027 [p-ERK/ERK]: 2.1460±0.2410 vs. 1.0765±0.2739, p = 0.015), suggesting that BBG improved CBDL-induced liver fibrosis via the non-Smad signaling pathway.

### Acute vascular response to P2X_7_ inhibition

#### Baseline body weight and hemodynamics

Table 3 reveals that the BW, MAP, HR, PP, and baseline perfusion pressure were not significantly different in CBDL rats before vehicle or selective P2X_7_ inhibitor oATP pre-incubation (all p>0.05).

**Table 3:** Baseline body weight and hemodynamic parameters of CBDL rats with vehicle or oATP preincubation.

	CBDL-vehicle *n* = 6	CBDL-oATP *n* = 8
**Baseline BW (g)**	401.5±13.2	391.3±9.3
**MAP (mm Hg)**	103.2±8.1	104.0±3.7
**HR (beats/min)**	334.8±11.4	318.0±12.5
**PP (mm Hg)**	15.5±1.3	14.9±1.3
**Baseline perfusion pressure (mm Hg)**	16.8±1.1	16.6±1.7

BW: body weight; MAP: mean arterial pressure; HR: heart rate;

PP: portal pressure. All *P*>0.05 between the two groups

#### The vascular response of portal-systemic collateral vascular bed

**Fig 6:**
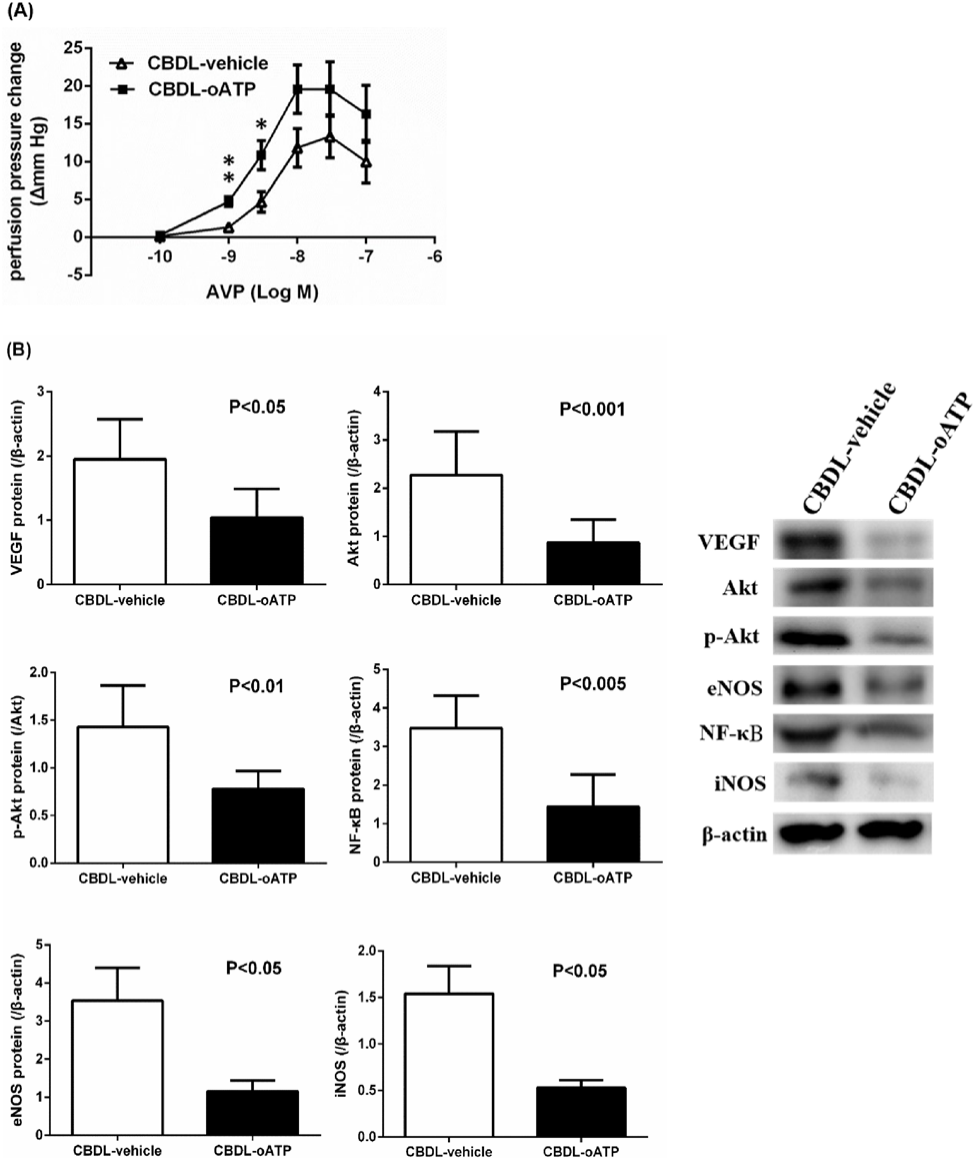
**(A) Concentration—response curves to arginine vasopressin (AVP) in the portal-systemic collateral vascular bed of CBDL rats pre-incubated with Krebs solution (vehicle) or oxidized ATP** (oATP, CBDL-vehicle: n = 6; CBDL-oATP: n = 7). AVP-induced perfusion pressure change in the CBDL-oATP group was higher than that in the CBDL-vehicle group. *p<0.05, **p = 0.001 vs. CBDL-vehicle group. **(B) Splenorenal shunt eNOS, iNOS, VEGF, Akt, p-Akt, and NF-κB protein expressions in common bile duct-ligated (CBDL) rats with vehicle or oxidized ATP (oATP) pre-incubation** (CBDL-vehicle: n = 5; CBDL-oATP: n = 6). oATP significantly down-regulated eNOS, iNOS, VEGF, Akt, p-Akt, and NF-κB expressions in splenorenal shunts of CBDL rats.

Fig 6(A) shows the perfusion pressure changes of portal-systemic collaterals in response to various concentrations of AVP in CBDL rats pre-incubated with vehicle or oATP. The vasoconstriction induced by AVP was significantly enhanced by oATP at the concentrations of 3 × 10^-9^ and 10^-8^ M (p = 0.001 and p = 0.023 vs. vehicle, respectively). The maximal pressure changes challenged with 125 mM KCl at the end of experiments were 19.5±1.7 (vehicle) and 22.1±4.3 (oATP) mm Hg respectively, not significantly different (p>0.05).

#### Splenorenal vascular eNOS, iNOS, and their related signaling molecule protein expressions

The splenorenal shunt NOS and the involving Akt and NF-κB signaling pathway protein expressions in CBDL rats are shown in Fig 6(B). As compared to vehicle, oATP pre-incubation markedly reduced VEGF, Akt, p-Akt, and eNOS expressions (CBDL-vehicle vs. CBDL-oATP [/β-actin]: VEGF: 1.9498±0.2791 vs. 1.0423±0.1998, p = 0.030; Akt: 2.2733±0.4515 vs. 0.8692±0.1813, p = 0.007. [p-Akt/Akt]: 1.4281±0.1943 vs. 0.7812±0.0757, p = 0.009; eNOS: 3.5360±0.8672 vs. 1.1529±0.2853, p = 0.015). In addition, oATP down-regulated NF-κB and iNOS expressions (CBDL-vehicle vs. CBDL-oATP [/β-actin]: NF-κB: 3.4797±0.3772 vs. 1.4390±0.3156, p = 0.002; iNOS: 1.5416±0.2979 vs. 0.5279±0.0846, p = 0.025).

## Discussion

A salient feature of portal hypertension is the formation of an extensive network of portal-systemic collateral vessels including the gastroesophageal varices, which may rupture with a high mortality rate [27]. Angiogenesis has been ascribed for the progression of collaterals [6]. In the current study, BBG, a P2X_7_ receptor antagonist as an “edible” blue dye significantly ameliorated the severity of portal-systemic shunting and mesenteric angiogenesis in CBDL rats, implying that P2X_7_ inhibition can be a candidate to control the complications associated with angiogenesis in cirrhosis. P2X_7_ has been noticed for its role in angiogenesis: P2X_7_ expression is increased in tumor-related angiogenesis [28]. Selective P2X_7_ antagonist, oATP, inhibited tumor angiogenesis via suppression of MMP-2, MMP-9, and VEGF [18]. Multiple factors are involved in angiogenesis: COX-1 up-regulation with neovascularization has been found in human ovarian cancer. Actually, selective COX-1 inhibition effectively suppressed angiogenesis [29]. COX-2 is also associated with angiogenesis in gastric cancer [30]. COX-2 down-regulation with siRNA inhibited angiogenic factor expressions in gastric cancer cell line [31]. An increased vascular area and surface in mice carrying activated PDGFRβ has also been demonstrated [32]. Intraocular antagonism of PDGF-BB significantly suppressed retinal and subretinal neovascularization [33]. A previous study also indicated that eNOS deficiency impaired angiogenesis in hind limb-ischemic mice compared with wild-type controls. Furthermore, impaired angiogenesis in eNOS^-/-^ mice was not improved by VEGF administration, implying that eNOS acts downstream of VEGF [34]. In line with the previous studies, BBG down-regulated mesenteric VEGF, VEGFR2, p-VEGFR2, PDGF, PDGFRβ, COX-1, COX-2, and eNOS expressions. Furthermore, BBG did not influence the angiogenic factors expressions in sham rats, suggesting that BBG ameliorates pathological angiogenesis in cirrhosis without influencing the relatively normal vasculature.

TGF-β is the major fibrogenic cytokine in liver fibrogenesis [35], which acts through Smad transcription factors family and results in extracellular matrix production. Smad2 and Smad3 proteins are phosphorylated after TGF-β induction, and then translocate into the nucleus where they regulate the transcription of target genes. Smad7, an inhibitor of Smad signaling, prevents phosphorylation of Smad2 and Smad3 proteins [36]. TGF-β signaling also takes actions via Smad-independent pathways, such as ERK/mitogen-activated protein kinase (MAPK) pathways. Increasing evidences indicate the involvement of MAPK signaling pathway in liver fibrogenesis. It has been found that ERK activation leads to HSC activation and proliferation, resulting in tissue remodeling and hepatic fibrosis [37]. In this study with rats affected by CBDL-induced liver cirrhosis, BBG significantly alleviated the severity of hepatic collagen deposition as shown by Sirius red staining. This is supported by the previous study that P2X_7_ antagonism reduced hepatic collagen formation and accumulation induced by chronic CCl_4_ treatment [38]. Furthermore, it is noteworthy that BBG obviously reduced hepatic TGF-β and ERK expressions without affecting Smad family protein expressions in CBDL rats, indicating that BBG might improve hepatic fibrosis by down-regulating the TGF-β-MAPK pathway and α-SMA. Likewise, the anti-fibrotic effect of P2X_7_ blockade in liver through suppression of TGF-β and α-SMA expression had been reported previously [38], suggesting the potential application of P2X_7_ inhibition in controlling liver fibrogenesis.

Leukocytes amplify the inflammatory response by generating pro-inflammatory cytokines such as TNF-α, IL-6, and IL-1β, along with the myofibroblasts activation elicited by PDGF and TGF-β [39]. In this study, we found that BBG significantly ameliorated liver fibrosis and down-regulated fibrosis-related pro-inflammatory cytokines IL-6, TNF-α, IL-1β, and PDGF. This is in line with a previous study that pharmacological antagonism or genetic deletion of P2X_7_ receptor reduced IL-1β and TNF-α in mice bladder tissue [40]. IL-1β release suppressed by selective P2X_7_ blockade [41] or P2X_7_ deletion [42] have been reported as well. Taken together, BBG reduces hepatic pro-inflammatory cytokines IL-6, TNF-α, PDGF, and IL-1β expressions, down-regulates TGF-β signaling pathway, and ameliorates liver fibrosis.

There is one issue to be addressed: are the aforementioned findings linked to the anti-inflammatory effect of P2X_7_ antagonism? We treated BDL rats since the 15^th^ day after operation, at which time point liver fibrosis rather than inflammation was the predominant histological finding [43]. The result showed that BBG was still able to ameliorate liver fibrosis. Taken the previous evidences into consideration, P2X_7_ activation enhanced TGF-β1 mRNA expression in type-2 rat brain astrocytes [44]. Furthermore, as compared with the wild type mice, there were less myofibroblasts, diminished collagen deposition, and decreased TGF-β expression in the renal interstitium of P2X_7_ knockout mice subjected to unilateral ureteral obstruction [45]. Solini et al. [46] have also demonstrated the importance of P2X_7_ activation on TGF-β secretion and mesangial matrix expansion induced by hyperglycemia. Another animal study also indicated the reduced collagen deposition and fibrotic pathology in lungs of bleomycin-administered P2X_7_ knockout mice [42]. To sum up, P2X_7_ antagonism may ameliorate fibrosis directly in rats with BDL-induced liver fibrosis. Nevertheless, it is well known that excessive inflammation initiates the cascade of fibrogenesis, so the amelioration of liver fibrosis via P2X_7_ antagonism-induced anti-inflammation is not to be neglected. For instance, Vesey et al. [47] demonstrated that the pro-inflammatory cytokine IL-1β promoted fibroblast proliferation and collagen production. Regarding the potential influence on necrosis, both liver-resident cells such as Kupffer cells, HSCs, and cells that are recruited in response to injury elicit pro-inflammatory signals that contribute to hepatocyte necrosis [48]. Therefore, the reduction of hepatocyte necrosis by P2X_7_ antagonism is considered to be mediated via anti-inflammation.

In the current study, pre-incubation of oATP, another selective P2X_7_ antagonist [49], markedly enhanced portal-systemic collateral responsiveness to AVP in CBDL rats and down-regulated splenorenal shunt iNOS and eNOS expressions. Chiao et al found that P2X_7_ receptors activation in the presence of LPS stimulated iNOS expression and NO production, which elicited vasodilation and was reversed by oATP [10]. It has also been noted that iNOS was induced by NF-κB activation [50]. Moreover, absence of P2X_7_ receptor reduced iNOS expression as well as NF-κB activation in lung parenchyma [41]. Compatible with the previous findings, acute oATP administration significantly reduced NF-κB and iNOS protein expressions in splenorenal shunts of CBDL rats. Regarding eNOS, P2X_7_ receptor activation involves an initial upstream mechanism of LPS-induced vascular dysfunction, which is associated with IL-1β-mediated eNOS activation [51]. Furthermore, P2X_7_ deficiency reduced eNOS protein expression [52]. VEGF also increases eNOS activity and NO generation through Akt activation [53]. Consistently, the current study showed that oATP decreased VEGF, Akt, p-Akt, and eNOS protein expressions in splenorenal shunts of CBDL rats. The acute down-regulation of iNOS, eNOS, and their related signaling molecules by P2X_7_ antagonism might be responsible, at least partly, for the improved collateral AVP vasoresponsiveness and contribute to a better therapeutic effect of vasoconstrictors in cirrhotic patients suffering from acute gastroesophageal variceal hemorrhage, though further investigation is required.

The reason why we used oATP instead of BBG for perfusion experiments is that BBG is a blue dye which stains the tubes and catheters of the *in situ* perfusion system. Nevertheless, the nontoxic and “edible” nature of BBG makes clinical application possible. Therefore, we used oATP to test the impacts of P2X_7_ antagonism in perfusion experiments but BBG in chronic *in vivo* treatment. The differential effects of chronic and acute P2X_7_ antagonism on mesenteric and splenorenal shunt eNOS expressions are also worth noting: Previous studies have indicated that an enhanced mesenteric eNOS expression contributed to mesenteric angiogenesis in CBDL rats [49]. Moreover, eNOS is a potent vasodilator [53]. Therefore, eNOS affects both angiogenesis and vasodilation. Actually, promising results are noted in the current study: mesenteric eNOS down-regulation by chronic P2X_7_ antagonism alleviated mesenteric angiogenesis and splenorenal shunt eNOS down-regulation by acute P2X_7_ antagonism enhanced collateral vasoconstrictive response to AVP.

The SMA flow was reduced by BBG in CBDL rats, which may be related to the ameliorated mesenteric angiogenesis. However, BBG did not significantly affect portal pressure. Portal pressure is determined by the net effects of portal inflow, intrahepatic resistance, and portal-systemic collateral vascular resistance. SMA, the major branch of the abdominal aorta, is responsible for about a half of portal venous blood inflow and regarded as the most dominant flow index in splanchnic circulation of portal hypertension [54]. Intrahepatic resistance is determined by dynamic and mechanical components, including enhanced vascular tone and cirrhotic change [55]. On the other hand, the portal-systemic collaterals develop with the aim to divert the stagnant portal blood flow, so the increased collateral vascular resistance may increase portal pressure. Our data show that BBG significantly lowered SMA flow, alleviated liver fibrosis, and reduced the severity of portal-systemic collaterals. Moreover, oATP enhanced the portal-systemic collateral vasoresponsiveness to vasoconstrictor, suggesting the potential of P2X_7_ inhibition to increase collateral vascular resistance. Taken together, portal pressure is not influenced by P2X_7_ inhibition in the current experimental setting. Nevertheless, the amelioration of portal-systemic shunting, mesenteric angiogenesis and improved collateral AVP responsiveness may be of clinical significance in alleviating gastroesophageal varices and in improving the treatment response to vasoconstrictors during acute hemorrhage. Furthermore, mean arterial pressure was not significantly influenced, suggesting that P2X_7_ inhibition is hemodynamically feasible for cirrhotic patients with pre-existing hyperdynamic circulation and systemic hypotension. BBG also significantly reduced ALT in CBDL rats, which is compatible with the anti-inflammatory nature of P2X_7_ antagonism [12–17].

To sum up, purinergic receptor inhibition, especially P2X_7_ receptor blockade, ameliorates liver fibrosis, mesenteric angiogenesis, severity of portal-systemic shunting, and improves the portal-systemic collateral vascular responsiveness in cirrhotic rats, suggesting the potential of purinergic receptor antagonism in controlling liver cirrhosis-related complications.
